# Potentially repurposable drugs for COVID-19 identified from SARS-CoV-2 Host Protein Interactome

**DOI:** 10.21203/rs.3.rs-30363/v1

**Published:** 2020-05-28

**Authors:** Kalyani B. Karunakaran, N. Balakrishnan, Madhavi Ganapathiraju

**Affiliations:** Indian Institute of Science; Indian Institute of Science; University of Pittsburgh

**Keywords:** interactome, network analysis, drug repurposing

## Abstract

We previously presented the protein-protein interaction network - the ‘HoP’ or the host protein interactome - of 332 host proteins that were identified to interact with 27 nCoV19 viral proteins by Gordon et al. Here, we studied drugs targeting the proteins in this interactome to identify whether any of them may potentially be repurposable against SARS-CoV-2. We studied each of the drugs using the BaseSpace Correlation Engine and identified those that induce gene expression profiles negatively correlated with SARS-associated expression profile. This analysis resulted in 20 drugs whose differential gene expression (drug versus normal) had an anti-correlation with differential expression for SARS (viral infection versus normal). These included drugs that were already being tested for their clinical activity against SARS-CoV-2, those with proven activity against SARS-CoV/MERS-CoV, broad-spectrum antiviral drugs, and those identified/prioritized by other computational re-purposing studies. In summary, our integrated computational analysis of the HoP interactome in conjunction with drug-induced transcriptomic data resulted in drugs that may be repurposable for COVID-19.

## Introduction

COVID–19 (Coronavirus Disease 2019) is an infectious virus outbreak which rapidly developed into a pandemic health crisis. The novel coronavirus (SARS-CoV–2/nCoV–19) is the causative agent of this disease.^1^ Several groups responded to the urgent need for effective therapeutics by leading systems-level efforts to identify drugs repurposable for COVID–19, through the lens of the virus-host protein interactome,^2^ and the interactomes of SARS-CoV–2-modulated host proteins^3^ and host proteins modulated by other human corona viruses such as SARS-CoV, MERS-CoV, HCoV–229E, and HCoV-NL63.^4^ Repurposing or finding alternate uses for approved drugs has proved to be a better strategy than *de novo* identification in terms of time and cost effectiveness.^5–7^

Discovery of therapeutic agents for infectious diseases in the past was largely serendipitous, and focused on screening and prioritizing drugs that target the viral system.^8^ Over the last few years, the focus has shifted towards computationally identifying drugs that could counter the virus attack A primary strategy is to repurpose drugs with the ability to revert the genes differentially expressed in the host upon viral infection to their normal levels; i.e. to revert the host transcriptional profile induced upon viral infection to its normal state.^8^ This “inverse genomic signature approach” involves identifying drugs that induce gene expression profiles negatively correlated with host-specific gene signatures induced by viral infection, and has been used to select candidates repurposable against influenza viruses and MERS-CoV.^9–11^ Availability of disease-associated and drug-induced transcriptomic profiles in online repositories such as NCBI GEO (Gene Expression Omnibus) and CMAP (Connectivity Map),^12,13^ allow these profiles to be compared using bioinformatics data analysis software suites such as the BaseSpace Correlation Engine.^14^ Changes in the host transcriptome induced by viral infection are also reflected in the host proteome, specifically as perturbations in the interaction networks of the host proteins. This complex network of protein-protein interactions (PPIs) called the ‘interactome’ has the potential to restrict viral replication in host cells, or conversely to be taken over by the virus for its replication.

We had previously presented the Host Protein Interactome (HoP Interactome) of 332 human proteins identified to interact with 27 SARS-CoV–2 viral proteins by Gordon et al.^2,15^. This interactome, consisting of 6,076 PPIs of the host proteins including 1,941 novel interactions predicted by HiPPIP, provided an integrated view on how host genes in various high throughput COVID–19 and SARS transcriptomic/proteomic studies are functionally linked.^15^ In this study, we identified drugs targeting the proteins in this interactome, and studied the correlation of the gene expression profiles induced by these drugs in various cell lines, with SARS/COVID-associated profiles observed in lung-derived (MRC5, Calu–3, NHBE and A549) cell lines, and in peripheral blood mononuclear cells (PBMCs) of SARS patients. Our work differs from previous efforts to identify drugs repurposable for COVID–19^2–4^ in that it considers the host protein interactome, and includes computationally predicted novel interactors of the host proteins, which may lead to identification of drugs that were hitherto not prioritized.

## Results

### Potentially Repurposable Drugs

We compared drug-induced versus SARS-associated differential expression using the BaseSpace Correlation Engine (previously called NextBio) (https://www.nextbio.com),^16,17^ to identify drugs for nCoV19. We compiled a list of 933 chemical compounds whose differential gene expression profile (drug versus no drug) were negatively correlated with at least one of the four SARS differential gene expression datasets (infected versus non-infected); the 4 SARS datasets we studied were: Calu–3 epithelial cells infected for 48 hours with SARS coronavirus versus mock infected cells (GSE17400), Calu–3 lung cells infected for 72 hours with SARS CoV Urbani versus mock infected cells (GSE37827), lung fibroblast MRC5 cells 24 hours post SARS coronavirus infection, high multiplicity of infection MOI versus mock infection (GSE56189) and peripheral blood mononuclear cells (PBMCs) from patients with SARS versus healthy subjects (GSE1739^18^). We also compiled a list of 381 chemical compounds with gene expression profiles negatively correlated with the profile induced in human bronchial epithelial (NHBE) and lung cancer (A549) cells infected with the SARS-CoV–2 strain USA-WA1/2020 (GSE147507^19^) Although in each case, there would be some genes that are differentially expressed in the same direction for both the drug and the disease (i.e., both cause some genes to overexpress, or both cause some genes to under express), the overall effect on the entire transcriptome would be an anti-correlation. A correlation score is generated by NextBio based on the strength of the overlap between the drug and disease datasets. Statistical criteria such as correction for multiple hypothesis testing are applied and the correlated datasets are then ranked by statistical significance. A numerical score of 100 is assigned to the most significant result, and the scores of the other results are normalized with respect to this top-ranked result.

Next, we identified 1,130 drugs that target at least one protein in the HoP interactome using WebGestalt.^20^ We used the ‘redundancy reduction’ feature provided by WebGestalt to prioritize drugs with highly significant overlaps with the interactome, while also capturing all the unique target gene sets. This feature used an affinity propagation algorithm which clusters sets of genes in the interactome targeted by specific drugs using Jaccard index as the similarity metric and identifies a ‘representative’ for each cluster (one drug and its targets), having the most significant p-value among all the gene sets in that cluster. This resulted in 209 drugs for further consideration. Given a class of drugs targeting the same set of proteins, this method ensures that only those individual drugs that target a statistically significant number of proteins in the interactome are prioritized for further analysis.

**Figure 1:**
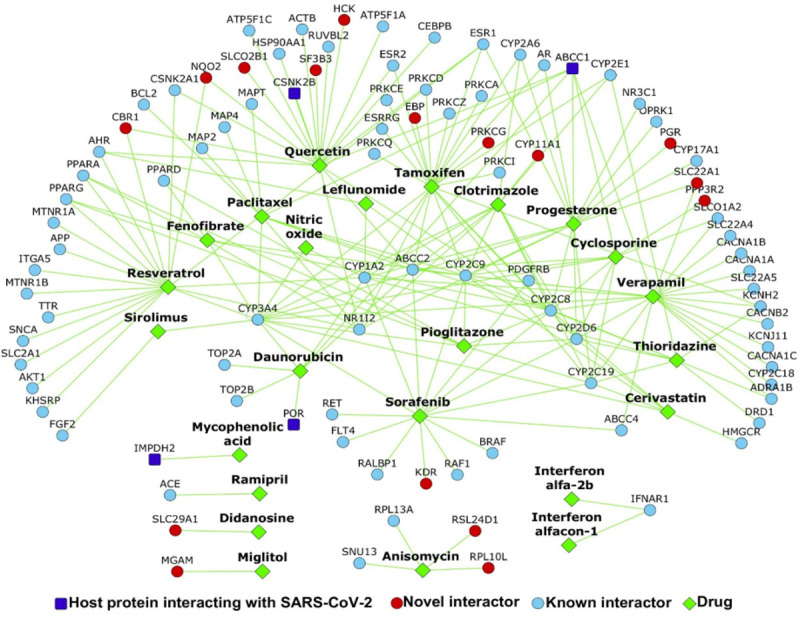
Repurposable drugs for COVID-19: The network shows the drugs (green color nodes) that target the proteins in the CoV-HP interactome. Host proteins are shown as dark blue nodes, their known interactors are light blue and novel interactors are red.

Fifty-six drugs were found in common to the above two analyses, i.e. these drugs not only targeted genes in the HoP interactome, but also induced gene expression profiles which are negatively correlated with that induced by SARS-CoV (Supplementary Table S1) and SARS-CoV–2 (Supplementary Table S2).. Thirteen drugs showed negative correlation with both expression profiles. Twenty-four of the fifty six have supporting evidence for biological relevance through clinical trial data and published literature (Figure 1) (in the list below, three drugs—cyclosporine, sorafenib and tamoxifen—have multiple evidences, and are shown italicized after 1^st^ occurrence):

- four drugs showed activity against SARS-CoV–2 in vitro (anisomycin, cyclosporine, sorafenib, tamoxifen)
- one chemical compound (nitric oxide) found here is already being tested against nCoV19 in clinical trials
- one drug (ramipril) belongs to the class of receptors targeted by nCoV19
- five drugs display antiviral activity in SARS or MERS infected cells line (cyclosporine, interferon alfacon–1, interferon alpha–2b, mycophenolic acid, resveratrol, sirolimus)
- three drugs (progesterone, quercetin, verapamil) are active against influenza viruses two drugs active against DNA viruses (sorafenib, daunorubicin, leflunomide), and
- eight drugs show activity against other RNA viruses (cerivastatin, clotrimazole, didanosine, fenofibrate, miglitol, paclitaxel, pioglitazone, tamoxifen, thioridazine)

8 drugs from our shortlist were independently identified or prioritized by other groups, namely: sirolimus (Zhou et al.^4^), leflunomide, quercetin and verapamil (Barabási et al.^3^), interferon alfa–2b, resveratrol, cyclosporine and mycophenolic acid (Li et al.^21^). Additionally, eight out of the 24 shortlisted drugs were also found among 127 broad-spectrum antiviral drugs active against 80 viruses (https://drugvirus.info/). These are cyclosporine, leflunomide, mycophenolic acid, sirolimus, sorafenib, tamoxifen, anisomycin and verapamil.

## Discussion

An integrated computational analysis of the interactome in conjunction with drug-induced transcriptomic data revealed 24 drugs that may be repurposable for COVID–19. These included drugs with proven *in vitro* activity against SARS-CoV–2, those that were already being tested for their clinical activity against SARS-CoV–2, those with proven activity against SARS-CoV/MERS-CoV, broad-spectrum antiviral drugs, and those identified/prioritized by other computational re-purposing studies.

### Information from literature supporting the shortlisted drugs

*Cyclosporine, sorafenib, tamoxifen and anisomycin* were identified to have inhibitory effects on SARS-CoV–2 in four independent cell-based assays.^22–25^ These drugs have also been shown to be effective against viruses similar to SARS-CoV–2 or other viruses. *Cyclosporine* in combination with interferon alpha reduced MERS-CoV replication and this reduction was associated with greater induction of interferon stimulated genes.^26^ *Sorafenib* has been shown to suppress the gene expression of HBV (hepatitis B virus) by inhibiting the JNK pathway, which constitutes FXR, a transcription factor that promotes HBV replication and gene expression.^27^ *Tamoxifen* has shown inhibitory effect against the vesicular stomatitis virus, whose effect in Vero cells has been correlated with activation of macrophages and an elevated interferon-I response.^28^ Anisomycin reduced the viral load of Zika virus in the blood of AG129 mice.^29^ Testing of *nitric oxide* gas inhalation therapy is already underway in COVID–19 patients (see www.ClinicalTrials.gov for ongoing clinical trials for nitric oxide). *Nitric oxide* is usually produced by phagocytes in response to interferon-γ. However, it is also rapidly produced in primary fibroblasts in response to viral dsRNA, especially in the absence of an intact interferon system, which has been noted in the case of nCoV19 infection.^30^ *Resveratrol* has shown anti-viral activity in MERS-infected Vero E6 (kidney epithelial) cells.^31^ It inhibited viral infection, prolonged the survival of the host cell after infection by downregulating virus-induced apoptosis and reduced the expression of the viral nucleocapsid protein, which is essential for viral replication.^31^ The mTOR inhibitor *sirolimus* reduced MERS-CoV infection by 60% in Huh7, a hepatocyte-derived cell line.^32^ *Mycophenolic acid* has been shown to inhibit papain-like protease of MERS-CoV.^33,34^ A significant lack of IFN-I and IFN-III (type I and III interferons) expression was noted in nCoV19 infected human bronchial epithelial and lung alveolar carcinoma cells.^19^ Prioritizing recombinant interferons (that are exogenously produced under laboratory conditions) as potential therapeutic options for COVID–19 may be essential in this scenario. *Interferon-alpha2b* in combination with ribavirin reduces viral replication, regulates the host response and improves clinical outcome in rhesus macaques infected with MERS-CoV.^35^ SARS-CoV infection in human bronchial epithelial Calu–3 cells has been shown to be inhibited by *interferon alfacon–1*.^36^ *Ramipril* is an inhibitor of the angiotensin converting enzyme (ACE1) which belongs to the class of entry receptors targeted by nCoV19. Current evidence shows that the spike protein of SARS-CoV–2 binds to ACE2 and not ACE1.^37^ Even though the two enzymes are coded by homologous genes, ACE1 inhibitors are incapable of acting on ACE2 and the physiological actions of ACE1 differ from that of ACE2 (vasoconstriction versus vasodilation). However, since these enzymes function in two counterbalancing arms of the renin-angiotensin system, more investigations are necessary to ascertain whether ACE1 has some (as yet) unidentified role in viral pathogenesis.^38^ *Quercetin* inhibits the infection of various influenza viruses including H1N1, H3N2 and H5N1, and it is suspected that this inhibition arises from their interaction with the viral HA2 (hemagglutinin) subunit.^39^ The calcium channel blocker *verapamil* inhibits the influenza virus late in their replication cycle, in Madin-Darby canine kidney cells and in murine pulmonary macrophages.^40^ *Progesterone* has been shown to promote faster recovery of female mice from influenza A virus infection by reducing pulmonary inflammation, repairing damaged lung epithelium and generally improving lung function.^41^ *Clotrimazole* inhibits the entry of recombinant vesicular stomatitis virus pseudotypes (arenavirus) into A549 human lung epithelial cells by targeting the membrane fusion mechanism of the virus.^42^ *Didanosine* is a nucleoside analogue and a reverse transcriptase inhibitor that is used to treat HIV/AIDS. In the host cell, *didanosine* is converted to dideoxyadenosine–5′-triphosphate (ddATP), whose incorporation into the viral DNA terminates DNA chain elongation, by preventing the formation of the 5′ to 3′ phosphodiester links.^43^ *Paclitaxel* inhibits the invasion of HIV–1 pseudovirus in TZM-bl cells (HeLa cell line).^44^ *Fenofibrate* has been shown to provide neuroprotection against Japanese encephalitis in a mouse model of viral infection by inducing the expression of genes that de-toxify pro-inflammatory leukotrienes, and by reducing microglial activation and the expression of chemokines and cytokines.^45^ *Cerivastatin* reduced the production of infectious Zika virus particles in Vero cells.^46^ Crystallographic analysis has shown that the anti-psychotic drug thioridazine directly interacts with the glycoprotein subunit of the Ebola virus.^47^ Although this interaction shows no direct pharmacological value against SARS-CoV–2, chlorpromazine, the parental compound of thioridazine, has shown activity against a range of viruses.^48^ *Leflunomide* has demonstrated anti-viral activity against CMV (cytomegalovirus) through inhibition of virion assembly.^49^ *Pioglitazone* showed anti-inflammatory activity against HIV in mice.^50^ *Daunorubicin* inhibited the replication of Hepatitis B virus in human hepatocyte NKNT–3/NTCP cells by activating innate immune response.^51^ *Miglitol* has been found to be active against hemorrhagic fever viruses.^52^

In this study, we shortlisted drugs potentially repurposable for COVID–19 based on the negative correlation of drug-induced versus disease-associated gene expression profiles. Although this approach has been validated in the past, it has several limitations. Drug associated expression profiles analyzed in this study were induced in several types of cell lines (including cancer cell lines) that may not be directly relevant to COVID–19 or SARS-CoV–2 infection. The effect of the proposed repurposable drugs should be studied in human bronchial epithelial cells and/or in human lung cancer cell lines, both of which were recently used to study host transcriptional response upon SARS-CoV–2 infection.^19^

In summary, we showed that drugs repurposable for COVID–19 can be identified from the host protein interactome based on gene expression profiles induced by the drug versus those associated with the disease. The dissemination of this list of repurposable drugs to the scientific community will enable clinical translation of these results.

## Methods

The list of chemical compounds whose gene expression profiles correlated negatively with four SARS datatsets and one COVID–19 dataset were compiled using the BaseSpace correlation software (https://www.nextbio.com) following the protocol described in prior work^17^ (List 1). The datasets considered were human bronchial epithelial (NHBE) and lung cancer (A549) cells infected with the SARS-CoV–2 strain USA-WA1/2020 (GSE147507^19^), Calu–3 epithelial cells infected for 48 hours with SARS coronavirus versus mock infected cells (GSE17400), Calu–3 lung cells infected for 72 hours with SARS CoV Urbani versus mock infected cells (GSE37827), lung fibroblast MRC5 cells 24 hours post SARS coronavirus infection, high MOI (3) versus mock infection (GSE56189) and peripheral blood mononuclear cells (PBMCs) from patients with SARS versus healthy subjects (GSE1739^18^). Next, we identified drugs that targeted at least one gene in in the HoP interactome using WebGestalt.^20^ After employing the ‘redundancy reduction’ feature in WebGestalt to reduce the search space of drugs, we were left with a fewer number of drugs (List 2). In this feature, an affi nity propagation algorithm clusters gene sets in the interactome targeted by specific drugs using Jaccard index as the similarity metric, and identifies a ‘representative’ for each cluster (one drug and its targets), having the most significant p-value among all the gene sets in that cluster. We then compared list 1 and list 2 to identify the drugs that not only target proteins in the interactome but are also negatively correlated with SARS/COVID–19.

List of drugs validated to be effective against SARS-CoV–2 in cell-based assays were obtained from the COVID–19 Gene and Drug Set Library (https://amp.pharm.mssm.edu/covid19/).^53^

The drug-protein interactome figure was created using Cytoscape.^54^

## Supplementary Material

Supplement
