# Supplementary material for: Potentially repurposable drugs for COVID-19 identified from SARS-CoV-2 Host Protein Interactome
Source: Res Sq. 2020 May 28:rs.3.rs-30363. Preprint. [Version 1] doi: 10.21203/rs.3.rs-30363/v1 (PMC7336709; doi:10.21203/rs.3.rs-30363/v1)
Supplement: Supplement [file SUPPLEMENTARYMATERIAL.docx]

SUPPLEMENTARY MATERIAL

**Supplementary Table S1. 51 drugs with expression profiles negatively correlated with SARS-associated profiles:** The correlation score is based on the strength of the overlap or enrichment between the two biosets. Additional statistical criteria such as correction for multiple hypothesis testing are applied and the correlated biosets are then ranked by statistical significance. A numerical score of 100 is assigned to the most significant result, and the scores of the other results are normalized with respect to the top-ranked result.

| Drug | Bioset 1 | Bioset 2 | Score (scaled negative correlations) | # Up in bioset 1 (P-val), Down in bioset 2 (P-val) | # Down in bioset 1 (P-val), Up in bioset 2 (P-val) |
| --- | --- | --- | --- | --- | --- |
| Alprenolol | Lung fibroblast MRC5 cells 24 hrs post SARS corona virus infection high MOI (3)_vs_mock infection | MCF7 cells + alprenolol, 14uM _vs_ DMSO vehicle | 100 | 228 (9.1E-9) | 74 (0.1079) |
| Chloramphenicol | Calu-3 lung cells_SARS Cov urbani infected 72 hrs_vs_mock-infected | Liver of Crj-CD(SD)IGS rats 24hr after 28d daily dose of 1000mg-kg chloramphenicol _vs_ 0mg-kg | 100 | 75 (2.5E-9) | 78 (0.0006) |
| Clotrimazole | Calu-3 lung cells_SARS Cov urbani infected 72 hrs_vs_mock-infected | Liver of rats + CLOTRIMAZOLE at 52mg-kg-d in corn oil by oral gavage 3d _vs_ vehicle | 100 | 119 (6.5E-11) | 189 (0.0003) |
| Didanosine | Calu-3 epithelial cells infected for 48 hrs with SARS corona virus_vs_mock-infected | Primary rat hepatocytes + didanosine at 50uM in DMSO 1d_vs_vehicle | 100 | 30 (2.2E-6) | 49 (0.0027) |
| Epinephrine | Lung fibroblast MRC5 cells 24 hrs post SARS corona virus infection high MOI (3)_vs_mock infection | Heart of rats + EPINEPHRINE at 0375mg-kg-d in saline by intravenous 5d _vs_ vehicle | 100 | 107 (2E-7) | 75 (1.8E-5) |
| Fenofibrate | Calu-3 lung cells_SARS Cov urbani infected 72 hrs_vs_mock-infected | HUVEC cells treated with fenofibrate for 18hr _vs_ untreated | 100 | 395 (4.2E-16) | 230 (3.7E-15) |
| Fenoprofen | Calu-3 lung cells_SARS Cov urbani infected 72 hrs_vs_mock-infected | Kidney of rats + FENOPROFEN at 52mg-kg-d in corn oil by oral gavage 1d _vs_ vehicle | 100 | 72 (5.2E-13) | 69 (0.0015) |
| Ifosfamide | Calu-3 epithelial cells infected for 48 hrs with SARS corona virus_vs_mock-infected | Rhabdomyosarcoma xenografts F2 generation treated with ICE-T _vs_ original patient tumor untreated | 100 | 451 (6E-20) | 1733 (1.5E-7) |
| Irinotecan | Lung fibroblast MRC5 cells 24 hrs post SARS corona virus infection high MOI (3)_vs_mock infection | MCF7 breast cancer cells treated 6hr with 5x IC50 of topo I inhibitor SN38 _vs_ untreated | 100 | 1153 (4.9E-47) | 500 (0.0017) |
| Isoniazid | PBMC from patients with SARS_vs_healthy subjects | Blood of TB patients infected with M. tuberculosis - post 2HRZE/4HR therapy _vs_ before therapy | 100 | 334 (6E-46) | 450 (4.5E-13) |
| Isradipine | Calu-3 epithelial cells infected for 48 hrs with SARS corona virus_vs_mock-infected | HL60 cells + isradipine, 10.8uM _vs_ DMSO vehicle | 100 | 40 (5.7E-9) | 61 (0.0229) |
| Nitric Oxide | Lung fibroblast MRC5 cells 24 hrs post SARS corona virus infection high MOI (3)_vs_mock infection | HCT116 colon cancer cells + NO 24hr _vs_ untreated control | 100 | 420 (7.3E-44) | 231 (8.2E-9) |
| Paclitaxel | Lung fibroblast MRC5 cells 24 hrs post SARS corona virus infection high MOI (3)_vs_mock infection | Mammary adenocarcinoma did not respond to 3wk carboplatin/paclitaxel treatment _vs_ untreated | 100 | 561 (1.5E-29) | 269 (1.4E-7) |
| Phenethyl isothiocyanate | Calu-3 lung cells_SARS Cov urbani infected 72 hrs_vs_mock-infected | Primary human hepatocytes + 25uM phenethyl isothiocyanate for 48hr _vs_ vehicle | 100 | 401 (2.8E-12) | 389 (0.0062) |
| Riluzole | Calu-3 lung cells_SARS Cov urbani infected 72 hrs_vs_mock-infected | PC3 cells + riluzole, 14.8uM _vs_ DMSO vehicle | 100 | 166 (6.5E-5) | 258 (2.4E-6) |
| Sorafenib | Calu-3 lung cells_SARS Cov urbani infected 72 hrs_vs_mock-infected | Hodgkins lymphoma HD-MYZ cell line - 10uM perifosine 5uM sorafenib treated 24hr _vs_ vehicle control | 100 | 309 (7.5E-14) | 378 (5.7E-16) |
| Terazosin | PBMC from patients with SARS_vs_healthy subjects | Heart of rats + TERAZOSIN at 657mg-kg-d in water by oral gavage 5d _vs_ vehicle | 100 | 39 (0.0228) | 29 (0.031) |
| Tetracycline | PBMC from patients with SARS_vs_healthy subjects | Hepatocytes of female donors treated 24hr with 1uM tetracycline _vs_ 0uM | 100 | 50 (0.0017) | 98 (8.5E-14) |
| Adalimumab | Calu-3 epithelial cells infected for 48 hrs with SARS corona virus_vs_mock-infected | Psoriasis lesional skin of adalimumab regimen responders - wk2 _vs_ wk0 | 100 | 90 (4.6E-40) | 67 (2.1E-5) |
| Cyclosporine | Calu-3 epithelial cells infected for 48 hrs with SARS corona virus_vs_mock-infected | Lesional skins of atopic dermatitis 5mg/kg/d CsA responders - treated 12wk _vs_ baseline | 100 | 386 (2.6E-61) | 1165 (6E-19) |
| Infliximab | Calu-3 epithelial cells infected for 48 hrs with SARS corona virus_vs_mock-infected | Ulcerative colitis colon 10mg/kg infliximab regimen - 8w _vs_ baseline | 100 | 170 (2.6E-54) | 693 (5.7E-54) |
| Prednisone | Calu-3 epithelial cells infected for 48 hrs with SARS corona virus_vs_mock-infected | Blood of dengue patients 2mg/kg prednisolone treated 3d - 1mo follow up _vs_ pre-treatment | 100 | 370 (1.4E-90) | 571 (2.1E-7) |
| Interferon alfacon-1 | Lung fibroblast MRC5 cells 24 hrs post SARS corona virus infection high MOI (3)_vs_mock infection | A549 lung adenocarcinoma cells treated 24hr with 500IU infergen _vs_ untreated | 100 | 150 (6.4E-5) | 68 (2.3E-10) |
| Interferon alfa-2b | PBMC from patients with SARS_vs_healthy subjects | Healthy whole blood - treated with IFNa-2b _vs_ not treated | 100 | 148 (5E-12) | 187(0.012) |
| Dacarbazine | Lung fibroblast MRC5 cells 24 hrs post SARS corona virus infection high MOI (3)_vs_mock infection | HL60 cells + dacarbazine, 22uM _vs_ DMSO vehicle | 100 | 127 (6.5e-16) | 69 (0.0162) |
| Tamoxifen | Lung fibroblast MRC5 cells 24 hrs post SARS corona virus infection high MOI (3)_vs_mock infection | Mammary epithelial cells 48hr with 10uM tamoxifen _vs_ DMSO | 94 | 343 (1.5E-26) | 95 (1.9E-8) |
| Sumatriptan | Lung fibroblast MRC5 cells 24 hrs post SARS corona virus infection high MOI (3)_vs_mock infection | Brain of rats + SUMATRIPTAN at 1100mg-kg-d in water by oral gavage 3d _vs_ vehicle | 93 | 117 (4.1E-7) | 87 (4.7E-6) |
| Nortriptyline | Lung fibroblast MRC5 cells 24 hrs post SARS corona virus infection high MOI (3)_vs_mock infection | MCF7 cells + nortriptyline, 13.4uM _vs_ DMSO vehicle | 91 | 216 (1.2E-5) | 107 (1.2E-6) |
| Quercetin | Lung fibroblast MRC5 cells 24 hrs post SARS corona virus infection high MOI (3)_vs_mock infection | MCF7 cells + quercetin, 11.8uM _vs_ DMSO vehicle | 91 | 520 (1.4E-32) | 104 (0.0018) |
| Resveratrol | Lung fibroblast MRC5 cells 24 hrs post SARS corona virus infection high MOI (3)_vs_mock infection | MCF7 cells + resveratrol, 17.6uM _vs_ DMSO vehicle | 91 | 237 (3.9E-15) | 159 (2.3E-5) |
| Cerivastatin | PBMC from patients with SARS_vs_healthy subjects | Kidney of rats + CERIVASTATIN at 7mg-kg-d in corn oil by oral gavage 3d _vs_ vehicle | 90 | 46 (2.5E-5) | 63 (0.0121) |
| Thioridazine | Lung fibroblast MRC5 cells 24 hrs post SARS corona virus infection high MOI (3)_vs_mock infection | PC3 cells + thioridazine, 9.8uM _vs_ DMSO vehicle | 89 | 323 (1.7E-9) | 105 (1.4E-5) |
| Mycophenolic acid | Lung fibroblast MRC5 cells 24 hrs post SARS corona virus infection high MOI (3)_vs_mock infection | MCF7 cells + mycophenolic acid, 12.4uM _vs_ DMSO vehicle | 87 | 329 (2.4E-6) | 142 (0.0142) |
| Granisetron | PBMC from patients with SARS_vs_healthy subjects | Liver of rats + GRANISETRON at 175mg-kg-d in water by oral gavage 3d _vs_ vehicle | 86 | 60 (0.0021) | 47 (0.3425) |
| Ticlopidine | Lung fibroblast MRC5 cells 24 hrs post SARS corona virus infection high MOI (3)_vs_mock infection | PC3 cells + ticlopidine, 13.4uM _vs_ DMSO vehicle | 85 | 306 (7.8E-6) | 83 (0.0001) |
| Dobutamine | Lung fibroblast MRC5 cells 24 hrs post SARS corona virus infection high MOI (3)_vs_mock infection | PC3 cells + dobutamine, 11.8uM _vs_ DMSO vehicle | 84 | 69 (0.0023) | 42 (9.4E-9) |
| Permethrin | Lung fibroblast MRC5 cells 24 hrs post SARS corona virus infection high MOI (3)_vs_mock infection | Neural 3D tissue constructs 16d - treated on d14 with 2.5uM permethrin for 2d _vs_ untreated | 81 | 277 (5.7E-20) | 168 (1.3E-10) |
| Sirolimus | Calu-3 epithelial cells infected for 48 hrs with SARS corona virus_vs_mock-infected | SKBR3 line (mammary adenocarcinoma overexpressing HER2) + rapamycin 24hr _vs_ vehicle | 71 | 54 (0.46) | 517 (5.7E-54) |
| Epirubicin | Calu-3 epithelial cells infected for 48 hrs with SARS corona virus_vs_mock-infected | Breast tumors post epirubicin cyclophosphamide paclitaxel gemcitabine herceptin_vs_baseline | 68 | 80 (1.8E-23) | 452 (1.4E-6) |
| Timolol | Calu-3 lung cells_SARS Cov urbani infected 72 hrs_vs_mock-infected | Heart of rats + TIMOLOL at 900mg-kg-d in water by oral gavage 5d _vs_ vehicle | 65 | 108 (1.7E-12) | 227 (2.2E-5) |
| Miconazole | Calu-3 lung cells_SARS Cov urbani infected 72 hrs_vs_mock-infected | HL60 cells + miconazole, 9.6uM _vs_ DMSO vehicle | 64 | 102 (0.0003) | 108 (0.0248) |
| Metyrapone | Calu-3 lung cells_SARS Cov urbani infected 72 hrs_vs_mock-infected | MCF7 cells + metyrapone, 17.6uM _vs_ DMSO vehicle | 62 | 162 (0.0004) | 88 (0.0096) |
| Nitrazepam | Lung fibroblast MRC5 cells 24 hrs post SARS corona virus infection high MOI (3)_vs_mock infection | Liver 310mg per kg Nitrazepam treated 3d _vs_ vehicle control | 56 | 117 (1.1E-8) | 84 (0.033) |
| Perhexiline | Calu-3 lung cells_SARS Cov urbani infected 72 hrs_vs_mock-infected | liver of male rat + PERHEXILINE 320mg per kg for 5d _vs_ vehicle | 52 | 103 (0.0073) | 223 (8E-7) |
| Staurosporine | PBMC from patients with SARS_vs_healthy subjects | Primary rat hepatocytes + STAUROSPORINE at 1.3uM in DMSO 1d _vs_ vehicle | 41 | 143 (0.0004) | 232 (0.0285) |
| Leflunomide | Lung fibroblast MRC5 cells 24 hrs post SARS corona virus infection high MOI (3)_vs_mock infection | MCF7 cells + leflunomide, 14.8uM _vs_ DMSO vehicle | 40 | 70 (0.0007) | 30 (0.0968) |
| Verapamil | PBMC from patients with SARS_vs_healthy subjects | HL60 cells + verapamil, 8.2uM _vs_ DMSO vehicle | 39 | 38 (0.0001) | 43 (0.0299) |
| Hydrocortisone | Calu-3 epithelial cells infected for 48 hrs with SARS corona virus_vs_mock-infected | HUVECS 1uM hydrocortisone 500U/ml IL1B 2500U/ml TNFα + 1250U/ml IFNγ 4 hr_vs_vehicle | 36 | 71 (0.1487) | 324 (0.0692) |
| Progesterone | Lung fibroblast MRC5 cells 24 hrs post SARS corona virus infection high MOI (3)_vs_mock infection | MCF7 cells + progesterone, 12.8uM _vs_ DMSO vehicle | 31 | 270 (2.6E-10) | 116 (0.0386) |
| Ramipril | Lung fibroblast MRC5 cells 24 hrs post SARS corona virus infection high MOI (3)_vs_mock infection | MCF7 cells + ramipril, 9.6uM _vs_ DMSO vehicle | 31 | 147 (1.7E-7) | 53 (0.0052) |
| Temazepam | Calu-3 lung cells_SARS Cov urbani infected 72 hrs_vs_mock-infected | Cerebrocortical cells from E16.5 mice treated 8hr - 0.5uM temazepam _vs_ DMSO | 27 | 11 (0.0534) | 25 (0.0338) |

**Supplementary Table S2. 18 drugs with expression profiles negatively correlated with COVID-associated profile:** The correlation score is based on the strength of the overlap or enrichment between the two biosets. Additional statistical criteria such as correction for multiple hypothesis testing are applied and the correlated biosets are then ranked by statistical significance. A numerical score of 100 is assigned to the most significant result, and the scores of the other results are normalized with respect to the top-ranked result.

| Drug | Bioset 1 | Bioset 2 | Correlation score (scaled negative correlations) | # Up in bioset 1 (P-val), Down in bioset 2 (P-val) | # Down in bioset 1 (P-val), Up in bioset 2 (P-val) |
| --- | --- | --- | --- | --- | --- |
| Didanosine | Bronchial epithelial NHBE and lung cancer A549 cells infected with SARS-CoV-2 strain USA-WA1/2020 | Primary rat hepatocytes + DIDANOSINE at 500uM in DMSO 1d _vs_ vehicle | 76 | 43 (3.2E-19) | 20 (0.1725) |
| Isoniazid | Bronchial epithelial NHBE and lung cancer A549 cells infected with SARS-CoV-2 strain USA-WA1/2020 | Blood of TB patients infected with M. tuberculosis - post 2HRZE/4HR therapy _vs_ before therapy | 75 | 268 (2.4E-47) | 580 (5.9E-9) |
| Epirubicin | Bronchial epithelial NHBE and lung cancer A549 cells infected with SARS-CoV-2 strain USA-WA1/2020 | Liver 2.7mg per kg Epirubicin treated 3d _vs_ vehicle control | 66 | 92 (1.4E-21) | 100 (0.0001) |
| Paclitaxel | Bronchial epithelial NHBE and lung cancer A549 cells infected with SARS-CoV-2 strain USA-WA1/2020 | Ovarian cancer OVISE cells + 10X IC50 concentration of paclitaxel for 24hr _vs_ untreated | 66 | 82 (4.2E-15) | 91 (0.0358) |
| Daunorubicin | Bronchial epithelial NHBE and lung cancer A549 cells infected with SARS-CoV-2 strain USA-WA1/2020 | Heart 3.25mg per kg Daunorubicin treated 1d _vs_ vehicle control | 65 | 40 (1.3E-14) | 34 (0.0004) |
| Rifapentine | Bronchial epithelial NHBE and lung cancer A549 cells infected with SARS-CoV-2 strain USA-WA1/2020 | Kidney of rats + RIFAPENTINE at 75mg-kg-d in corn oil by oral gavage 1d _vs_ vehicle | 64 | 24 (0.0117) | 77 (2.2E-7) |
| Ticlopidine | Bronchial epithelial NHBE and lung cancer A549 cells infected with SARS-CoV-2 strain USA-WA1/2020 | Liver of Crj-CD(SD)IGS rats 24hr after 14d daily dose of 300mg-kg ticlopidine _vs_ 0mg-kg | 63 | 59 (2.9E-15) | 59 (0.2386) |
| Ifosfamide | Bronchial epithelial NHBE and lung cancer A549 cells infected with SARS-CoV-2 strain USA-WA1/2020 | Heart of rats + IFOSFAMIDE at 143mg-kg-d in saline by oral gavage 5d _vs_ vehicle | 61 | 34 (2.6E-15) | 93 (0.0091) |
| Quercetin | Bronchial epithelial NHBE and lung cancer A549 cells infected with SARS-CoV-2 strain USA-WA1/2020 | Hep G2 hepatocarcinoma cell line cultured for 24hr with 10nM quercetin _vs_ 0.5% DMSO | 60 | 25 (0.0016) | 233 (1.7E-12) |
| Resveratrol | Bronchial epithelial NHBE and lung cancer A549 cells infected with SARS-CoV-2 strain USA-WA1/2020 | AML THP-1 cells 24hr Mycobacterium tuberculosis infected - with 100uM resveratrol _vs_ without | 58 | 162 (2.8E-23) | 293 (3.1E-5) |
| Tetracycline | Bronchial epithelial NHBE and lung cancer A549 cells infected with SARS-CoV-2 strain USA-WA1/2020 | Primary rat hepatocytes + TETRACYCLINE at 520uM in DMSO .67d _vs_ vehicle | 58 | 19 (0.001) | 174 (6.2E-7) |
| Pioglitazone | Bronchial epithelial NHBE and lung cancer A549 cells infected with SARS-CoV-2 strain USA-WA1/2020 | Heart of rats + PIOGLITAZONE at 1500mg-kg-d in corn oil by oral gavage 5d _vs_ vehicle | 57 | 15 (0.343) | 91 (7.5E-7) |
| Chloramphenicol | Bronchial epithelial NHBE and lung cancer A549 cells infected with SARS-CoV-2 strain USA-WA1/2020 | Liver of Crj-CD(SD)IGS rats 24hr after 28d daily dose of 1000mg-kg chloramphenicol _vs_ 0mg-kg | 55 | 39 (5E-09) | 23 (0.0535) |
| Permethrin | Bronchial epithelial NHBE and lung cancer A549 cells infected with SARS-CoV-2 strain USA-WA1/2020 | Neural 3D tissue constructs 16d - treated on d14 with 2.5uM permethrin for 2d _vs_ untreated | 55 | 18 (0.4565) | 157 (5.5E-5) |
| Miglitol | Bronchial epithelial NHBE and lung cancer A549 cells infected with SARS-CoV-2 strain USA-WA1/2020 | Osteosarcoma U-2 OS cells treated 12hr with 1000nM miglitol _vs_ DMSO | 45 | 3 (0.0366) | 31 (8.4E-6) |
| Nortriptyline | Bronchial epithelial NHBE and lung cancer A549 cells infected with SARS-CoV-2 strain USA-WA1/2020 | Primary rat hepatocytes + NORTRIPTYLINE at 70uM in DMSO 1d _vs_ vehicle | 42 | 93 (2.7E-8) | 392 (0.0048) |
| Nitrazepam | Bronchial epithelial NHBE and lung cancer A549 cells infected with SARS-CoV-2 strain USA-WA1/2020 | Liver of rats + NITRAZEPAM at 310mg-kg-d in CMC by oral gavage 3d _vs_ vehicle | 41 | 22 (0.0471) | 119 (0.0016) |
| Anisomycin | Bronchial epithelial NHBE and lung cancer A549 cells infected with SARS-CoV-2 strain USA-WA1/2020 | PC3 cells + anisomycin, 15uM _vs_ DMSO vehicle | 41 | 75 (0.2238) | 281 (3.5E-5) |
